# Scientific Journal Editing in Challenging Times

**Published:** 2025-03

**Authors:** Iva Grabarić Andonovski, Zrinka Pongrac Habdija

**Affiliations:** Food Technology and Biotechnology journal; University of Zagreb Faculty of Food Technology and Biotechnology, Pierottijeva 6, 10000 Zagreb, Croatia

Last year, on 15 June, after his 67th birthday, we lost our dear Editor-in-Chief of the Food Technology and Biotechnology journal, Vladimir Mrša, *professor emeritus*. He led the journal for 15 years, and we became more friends than colleagues. Our almost daily office routine was to plan journal issues and future improvements, discuss the manuscripts’ status and research integrity issues over a cup of coffee. Although he was seriously ill in the last two years, Vlado (as everyone called him) was very active, not only as Editor-in-Chief, but also as the former President of the Croatian Association for Scholarly Communication (*1*). Our discussions always included the Association activities, such as the organisation of the PUBMET Summer School and Conference (*2*), webinars, panel discussions, and similar. Vlado was a modern *homo universalis*, with broad interests and vast knowledge about science, arts and technology, a type of person whom you cannot easily replace, which is also evident from the published *In memoriam* (*3*), written by his colleagues from the Laboratory for Biochemistry of the University of Zagreb Faculty of Food Technology and Biotechnology.

There are some guidelines on how to manage journals in times of crisis, such as war (*4*, *5*) or pandemic (*6*), but no one teaches you how to manage the journal in times of loss. There is no formal education for editors in Croatia, and most editors are volunteers who dedicate their time and efforts to managing the journal, a fact that must be pointed out, since it is easily neglected. It takes years to master the job, so it is very hard to replace someone who is leading the journal.

Nevertheless, with the help of our Deputy Editors, we are continuing our work. In the previous year, 528 manuscripts were submitted to the Food Technology and Biotechnology journal, of which 353 (approx. 67 %) were rejected by the Editor or Deputy Editors due to the lack of novelty, insufficient quality or incoherent presentation, or because the topic was outside the scope of the journal. An additional 41 were rejected based on the opinion of the Field editor, and 34 were cancelled or withdrawn by the authors. Of the remaining manuscripts, 33 were accepted for publication after the review, 33 were rejected and 40 manuscripts are still under evaluation. Average time for initial triage by the Editor and Deputy Editors was 14 days, for the Field editors’ evaluation 18 days, and for obtaining the reviews 124 days. The average time to make the decision was 41 days (for all manuscripts, accepted or rejected), but it took 277 days on average for a manuscript to be accepted for publication, mostly due to the numerous revisions (for some manuscripts up to 9). On average, we have obtained 2.4 reviews per paper (with an average of 9 reviewers assigned to the manuscript), and 269 Field editors and reviewers were involved in the process. In 2024 we published 46 scientific or review papers, one viewpoint, an editorial and an *In memoriam*, with a total of 464 pages.

The journal Impact Factor (*7*) is currently 2.3 (new one will be released in June), which positions the journal in Q3 (100/173 journals in Food science and technology and 110/174 journals in Biotechnology and applied microbiology). The 5-year JIF is 3.8 and the Journal Citation Indicator (JCI) is 0.46. According to the SCImago Journal & Country Rank (*8*), the journal is in Q2 (SJR=0.59), with H-index of 84 and CiteScore of 3.7 (CiteScoreTracker is 4.3).

We have updated our instructions to authors, including recommendations for the use of AI, which are now in line with our publisher’s guidelines (*9*). Additionally, our Open Access policy is now part of the publisher’s Open Science Policy (*10*). Starting from issue 2/2024, we are requiring authors to submit graphical abstracts along with manuscripts, and we have provided guidelines and tips for their preparation on the journal website. Graphical abstracts are not only published online and used to promote articles on social networks, but some of them have also been featured on the journal cover page. We are currently developing new publishing regulations to allow for more adaptable workflows and enable more frequent rotation of the journal board members and Editor-in-Chief. You will be notified as soon as these changes are implemented.

We are planning two Special Issues for 2025. One is entitled ‘Turning agricultural waste into useful biochemicals and biofuels through biochemical engineering and biotechnology processing’, with Guest editor Dr Lim Jun Wei from Universiti Teknologi PETRONAS, Malaysia, and it will provide our readers with interesting solutions to the waste problem in the food industry. The other Special issue is planned in honour of Vlado. It will bring together the colleagues he worked with throughout his career and left an imprint on them all.

We hope to successfully continue his legacy by editing our diamond open access journal to the highest quality standards.
